# Triglyceride-glucose index levels in patients with Klinefelter syndrome and its relationship with endothelial dysfunction and insulin resistance: a cross-sectional observational study

**DOI:** 10.20945/2359-3997000000594

**Published:** 2023-01-25

**Authors:** Ibrahim Demirci, Cem Haymana, Burcu Candemir, Bagdagul Yuksel, Mithat Eser, Coskun Meric, Safak Akin, Nese Ersoz Gulcelik, Alper Sonmez

**Affiliations:** 1 University of Health Sciences Gulhane Training and Research Hospital Department of Endocrinology and Metabolism Ankara Turkey Department of Endocrinology and Metabolism, Gulhane Training and Research Hospital, University of Health Sciences, Ankara, Turkey.; 2 Health Sciences University Gulhane Faculty of Medicine Department of Endocrinology and Metabolism Ankara Turkey Department of Endocrinology and Metabolism, Gulhane Faculty of Medicine, Health Sciences University, Ankara, Turkey.

**Keywords:** Klinefelter, TyG Index, ADMA, insulin resistance, hypogonadism

## Abstract

**Objectives:**

This study aimed to investigate the triglyceride-glucose (TyG) index, which is a simple surrogate marker of insulin resistance that is associated with various cardiometabolic diseases, in patients with Klinefelter syndrome (KS).

**Subjects and methods:**

A total of 30 patients with KS (mean age: 21.53 ± 1.66 years) and 32 healthy controls (mean age: 22.07 ± 1.01 years) were included in the study.The clinical and laboratory parameters,TyG index, asymmetric dimethylarginine (ADMA) level, homeostatic model assessment of insulin resistance (HOMA-IR) score, and high-sensitivity C-reactive protein level were measured in patients with KS and healthy subjects.

**Results:**

Patients with KS had higher HOMA-IR score (p = 0.043), ADMA levels (p < 0.001), and TyG index (p = 0.031) and lower high-density lipoprotein cholesterol levels (p < 0.001) than healthy subjects. TyG index was positively correlated with plasma ADMA (r = 0.48, p < 0.001) and HOMA-IR (r = 0.36, p = 0.011). Multivariate analyses showed that total testosterone level (β = −0.44, p = 0.001) and TyG index (β = 0.29, p = 0.045) were independent determinants of plasma ADMA levels.

**Conclusion:**

Patients with KS had higher TyG indices than healthy subjects. Moreover, TyG index was independently associated with endothelial dysfunction in patients. TyG index may be a practical and useful measure to show the increased endothelial dysfunction in patients with KS.

## INTRODUCTION

Klinefelter syndrome (KS) is a sex-chromosome disorder that is characterized by hypergonadotropic hypogonadism and represents the most common genetic cause of male infertility (1). The most commonly observed characteristics of patients with KS are eunuchoid body proportion, small hyalinized testes, azoospermia, infertility, and learning disabilities (1). In addition, the risk of cardiometabolic disorders and obesity are increased in patients with KS (2, 3).

The rates of morbidity and mortality are significantly elevated in patients with KS (4, 5). Cardiovascular diseases are among the most important causes for increased morbidity and mortality rates (6). Although environmental factors, chromosomal aberrations, and especially low testosterone levels play important roles in increasing the frequency of cardiovascular mortality, the mechanism underlying this relationship remains to be elucidated. In this regard, we recently demonstrated increased insulin resistance and impaired endothelial function in patients with KS (7).

The triglyceride-glucose (TyG) index, which is calculated using fasting blood glucose (FBG) and triglyceride (TG) levels, is a simple surrogate marker for predicting insulin resistance (8). Several studies have shown that the TyG index is also associated with increased risk of cardiovascular diseases, type 2 diabetes mellitus, and metabolic syndrome (9–11).

The identification of easily accessible and reliable markers has great clinical importance in predicting cardiovascular risk among patients with KS. TyG index may be a useful measure in this regard. However, there have been no data regarding TyG index in patients with KS. The aim of this study was to investigate whether TyG indices increase in patients with KS compared with those in healthy individuals and whether the index is related to endothelial dysfunction, insulin resistance, and inflammation.

## SUBJECTS AND METHODS

### Study population and design

This cross-sectional study included 30 male patients with KS who visited our outpatient clinic. Data for analysis were obtained from the database on hypogonadism patients from the Department of Endocrinology and Metabolism at our tertiary center. Thirty male subjects matched for age and body mass index (BMI) from the same database were enrolled as healthy controls. We included patients with a verified KS karyotype (all subjects included were 47, XXY) and who did not previously undergo testosterone replacement. The exclusion criteria were as follows: age < 18 years, acute infection, malignancy, any visceral organ dysfunction, nutritional derangements, clinical history of cardiovascular disease, cerebrovascular disease, use of drugs such as antidiabetic or lipid- lowering medications, or individuals without complete clinical data. All patients read and signed written informed consent forms. In addition, the local ethics committee of the Kecioren Training and Research Hospital, Turkey (27.03.2013/236), approved the study protocol, as defined in our previous study (7). A part of these data has also been used for a previous publication on KS (7). This study was registered at ClinicalTrials.gov (NCT05014997).

All anthropometric measurements were performed with all patients wearing only their underwear. Waist circumference (WC) was measured at the midline between the inferior costal margin and iliac crest and recorded in centimeters. For BMI calculation (kg/m^2^), the weight in kilograms was divided by the square of the height in meters. All patients with KS demonstrated low or normal serum total testosterone level and high gonadotropin (follicle stimulating hormone [FSH] and luteinizing hormone [LH]) levels and had karyotypes confirming the diagnosis of KS. The pituitary functions of patients were evaluated using appropriate methods.

### Sample collection and laboratory measurements

Venous blood samples were obtained between 08:00 and 09:00 am following overnight fasting, centrifugated (at 4,000 rpm for 15 min), and stored at −80 °C until assay. The FBG, total cholesterol (TC), TG, and high-density lipoprotein cholesterol (HDL-C) levels were measured by the enzymatic colorimetric method. The serum high-sensitivity C-reactive protein level was measured by immunoturbidimetric method (Olympus AU2700, Hamburg, Germany). The low-density lipoprotein cholesterol (LDL-C) level was calculated using the Friedewald equation (12). Total testosterone, FSH, LH, and serum basal insulin levels were evaluated using commercial kits, as previously reported (7). Homeostasis model assessment of insulin resistance (HOMA-IR) was calculated using the following equation: fasting insulin (mU/L) × fasting glucose (mg/dL)/405 (13). TyG index was calculated using the following formula: ln (fasting TG [mg/dL] × FBG [mg/dL]/2) (8). Plasma asymmetric dimethylarginine (ADMA) level was measured using validated, commercialized enzyme- linked immunoassay kits (Immundiagnostik, Germany).

### Statistical analyses

Data were expressed as means ± standard deviations for continuous variables or as percentages for categorical variables. Kolmogorov-Smirnov and Levene's tests were used for assessing normality. Independent sample *t*-test was used for comparisons among continuous variables and Chi-square test for categorical variables. Relationships between ADMA and HOMA-IR levels and clinical and biochemical parameters were evaluated using Pearson correlation coefficients. Multivariable logistic regression analysis was performed to assess the independent predictors of ADMA and HOMA-IR. Variables with significant univariate association with outcomes and variables that could be potential predictors were included in multivariate regression. Two-sided p values of ≤0.05 were considered statistically significant. SPSS 21.0 package program (SPSS Inc., ver. 25.0) was used for all statistical analyses.

## RESULTS

A total of 30 patients with KS (mean age: 21.53 ± 1.66 years) and 30 healthy controls (mean age: 22.07 ± 1.01 years) were included in the study. The baseline demographic and biochemical characteristics of patients and controls are shown in Table 1. The patient and control groups were similar in terms of age and BMI. There was no significant differences between WC, systolic blood pressure, diastolic blood pressure, FBG, TC level, LDL-C and hs-CRP level in the groups. As expected, FSH and LH levels were higher and total testosterone levels were lower (p < 0.001 for all) in patients with KS than in healthy controls. Moreover, HOMA-IR score (p = 0.043), ADMA level (p < 0.001), and TyG index (p = 0.031) were higher and HDL-C levels were lower (p < 0.001) in patients with KS.

**Table 1:** Demographic and laboratory parameters of patients with Klinefelter syndrome and healthy controls

Variables (mean ± SD)	Patients (n = 30)	Healthy controls (n = 30)	p
Age (year)	21.53 ± 1.66	22.07 ± 1.01	0.138
BMI (kg/m^2^)	23.25 ± 3.96	24.40 ± 2.52	0.205
WC (cm)	89.81 ± 13.19	85.63 ± 6.65	0.133
SBP (mmHg)	118.48 ± 10.74	123.73 ± 11.23	0.084
DBP (mmHg)	69.24 ± 8.84	68.82 ± 10.52	0.876
FBG (mg/dL)	83.31 ± 8.38	68.82 ± 10.52	0.163
Total-C (mg/dL)	160.66 ± 28.29	154.23 ± 29.99	0.437
LDL-C (mg/dL)	96.70 ± 23.49	89.27 ± 27.58	0.229
HDL-C (mg/dL)	40.83 ± 7.79	49.46 ± 8.75	**0.001**
TG (mg/dL)	123.48 ± 99.68	77.59 ± 32.77	**0.044**
FSH (mIU/mL)	52.02 ± 17.25	2.86 ± 1.99	**<0.001**
LH (mIU/mL)	28.86 ± 12.42	4.16 ± 1.58	**<0.001**
Total testosterone (ng/dL)	204.81 ± 151.34	503.02 ± 154.26	**<0.001**
Insulin (µU/mL)	14.23 ± 7.82	10.12 ± 10.05	0.142
HOMA-IR	3.39 ± 2.88	1.72 ± 2.74	**0.043**
hs-CRP (mg/L)	1.75 ± 2.52	0.89 ± 0.86	0.082
ADMA (µmol/L)	0.73 ± 0.21	0.49 ± 0.17	**<0.001**
TyG Index	8.36 ± 0.57	8.02 ± 0.48	**0.031**

ADMA: asymmetric dimethylarginine; BMI: body mass index; DBP: diastolic blood pressure; FBG: fasting blood glucose; FSH: follicle stimulating hormone; HDL: high-density lipoprotein cholesterol; HOMA-IR: Homeostatic Model Assessment for Insulin Resistance; hs-CRP: high-reactive C reactive protein; LDL-C: low-density lipoprotein cholesterol; LH: luteinizing hormone; SBP: systolic blood pressure; TG: triglycerides; Total-C: total cholesterol; TyG Index: Triglyceride to Glucose Index; WC: waist circumference.

**Figure 1:**
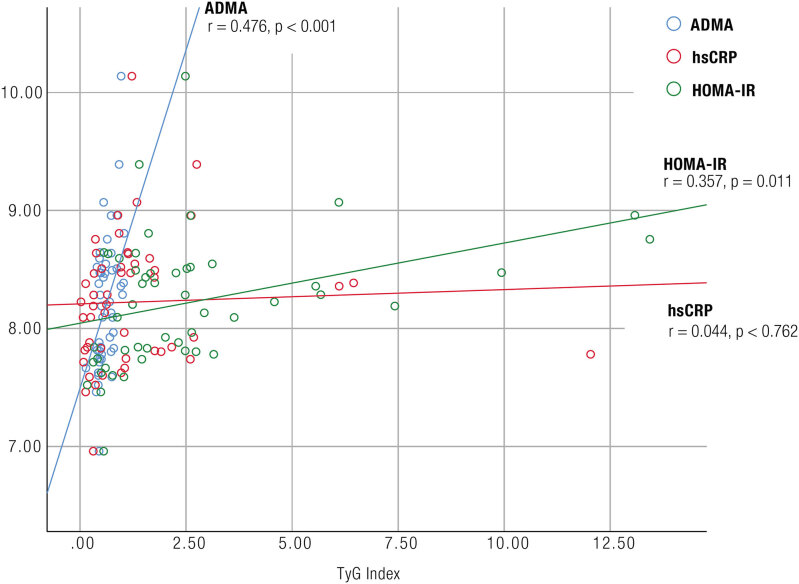
Scatter plot diagram of the correlation between TyG index and asymmetric dimethylarginine (ADMA), homeostatic model assessment-insulin resistance (HOMA-IR) and high sensitivity C-reactive protein (hs-CRP) levels.

In the correlation analysis, ADMA levels were positively correlated with WC (r = 0.31, p = 0.017), TG level (r = 0.44, p = 0.001), and TyG index (r = 0.48, p < 0.001) and were negatively correlated with total testosterone (r = −0.48, p < 0.001) and HDL-C (r = −0.42, p = 0.002) levels. HOMA-IR levels were positively correlated with TyG index (r = 0.36, p = 0.011) and negatively with HDL-C (r = -0.32, p = 0.022) levels (Table 2 and Figure 1).

**Table 2:** Correlation of ADMA level, HOMA-IR score, and clinical and biochemical parameters

	ADMA		HOMA-IR	
	r	p	r	p
BMI (kg/m^2^)	0.065	0.630	−0.028	0.849
WC (cm)	0.311	**0.017**	0.148	0.309
FPG (mg/dL)	−0.206	0.144	0.169	0.231
Total-C (mg/dL)	0.049	0.733	0.117	0.418
Triglyceride (mg/dL)	0.439	**0.001**	0.197	0.170
HDL-C (mg/dL)	−0.418	**0.002**	−0.323	**0.022**
LDL-C (mg/dL)	0.016	0.914	0.122	0.399
Insulin (µU/mL)	0.188	0.140	0.991	**<0.001**
SBP (mmHg)	−0.186	0.167	−0.043	0.772
DBP (mmHg)	0.024	0.866	−0.112	0.465
Total testosterone (ng/dL)	−0.481	**<0.001**	−0.203	0.153
TyG Index	0.476	**<0.001**	0.357	**0.011**
hs-CRP	0.125	0.330	0.023	0.873

ADMA: asymmetric dimethylarginine; HOMA-IR: Homeostatic Model Assessment for Insulin Resistance; BMI: body mass index; WC: waist circumference; FBG: fasting blood glucose; Total-C: total cholesterol; HDL-C: high-density lipoprotein cholesterol; LDL-C: low-density lipoprotein cholesterol; SBP: systolic blood pressure; DBP: diastolic blood pressure; TyG Index: Triglyceride to Glucose Index; hs-CRP: high-reactive C reactive protein.

Multiple regression analysis revealed that total testosterone level (β = −0.44, p = 0.001) and TyG index (β = 0.29, p = 0.045) were the independent determinants of plasma ADMA levels (Table 3 and Table 4, respectively).

**Table 3:** Linear regression of asymmetric dimethylarginine (ADMA) as the dependent variable

	Unstandardized coefficients		Standardized coefficient (β)	t	p
	β	SE			
WC	0.002	0.003	0.102	0.765	0.448
Total testosterone	<0.001	<0.001	−0.444	−3.602	**0.001**
TyG Index	0.116	0.056	0.288	2.064	**0.045**

TyG Index: Triglyceride to Glucose Index; WC: waist circumference.

**Table 4:** Linear regression of homeostatic model assessment of insulin resistance (HOMA-IR) as the dependent variable

	Unstandardized coefficients		Standardized coefficient (β)	t	p
	β	SE			
HDL-C	−0.061	7.942	−0.194	−1.263	0.213
TyG Index	1.374	0.809	0.261	1.699	0.096

HDL-C: high-density lipoprotein cholesterol; HOMA-IR: Homeostatic Model Assessment for Insulin Resistance; TyG Index: Triglyceride to Glucose Index.

## DISCUSSION

The results of present study show that patients with KS have higher HOMA-IR score, ADMA level, and TyG index than healthy controls. In addition, TyG index is significantly correlated with plasma ADMA level and HOMA-IR score and can be considered as an independent predictor of plasma ADMA levels. These results imply that TyG index can be a practical and useful measure for assessing endothelial dysfunction in patients with KS. To our knowledge, this is the first study to show the relationship of TyG index with endothelial dysfunction and insulin resistance in patients with KS.

Cardiovascular morbidity and mortality are common in KS (5, 6). However, the pathophysiology of increased mortality from cardiovascular diseases is uncertain. Previous studies showed increased prevalence of several cardiovascular risk factors, such as insulin resistance, diabetes mellitus, and abdominal obesity in KS (14, 15). The classical pathways leading to atherosclerosis, such as endothelial dysfunction, insulin resistance, and chronic subclinical inflammation, may play an important role in the increased cardiometabolic risk among patients with KS. Endothelium-derived mediators such as nitric oxide (NO) play a pivotal role in the pathogenesis of atherosclerotic diseases. Decreased NO levels may lead to endothelial dysfunction and thus increase the risk of cardiometabolic diseases. ADMA inhibits NO synthase, resulting in decreased NO levels and ultimately leading to endothelial dysfunction. ADMA is a well-known surrogate marker of endothelial dysfunction, and its high levels have been reported in many cardiometabolic diseases such as hypertension, type 2 diabetes, dyslipidemia, and chronic renal disease (16, 17). Similarly, insulin resistance is an important factor in the pathogenesis of atherosclerosis (18). As in our previous study (7), here, we demonstrated that plasma ADMA level and HOMA-IR score were significantly higher in patients with KS than in healthy controls. However, these surrogate markers of endothelial dysfunction and insulin resistance that are generally used in scientific studies are not practical and widely available to indicate increased cardiometabolic risk among patients with KS.

TyG index, which can be calculated practically using FBG and TG levels, was considered as an alternative marker of insulin resistance by Guerrero-Romero and cols. (19). Several studies have reported that the TyG index reflects insulin resistance better than HOMA-IR score (20). Increased TyG index has been reported in various cardiometabolic diseases such as coronary artery disease, hypertension, type 2 diabetes, and obesity, all of which are also considerably increased in patients with KS (11, 21–24). In addition to these reports, we showed for the first time that TyG index increases in patients with KS than in healthy subjects. Although there are no studies in patients with KS, there are only two studies assessing TyG indices in patients with hypogonadism. In one of these studies, Zhang and cols. reported higher TyG indices in patients with hypogonadism (25). However, the etiology of hypogonadism was not specified, and the study included elderly population. In the second study, we showed increased TyG indices in young and treatment-naïve patients with congenital hypogonadotropic hypogonadism (26). In the present study, we also showed that TyG index was an independent predictor of plasma ADMA level and HOMA-IR score. Similarly, together with the total testosterone levels, TyG index was found to be an independent predictor of plasma ADMA levels. Although there was a correlation between HOMA-IR score and TyG index, we could not find a relationship between these parameters in the regression analyses. Despite the strong association between the TyG index and HOMA-IR scores in the literature, the most important reason this relationship was not observed in the present study may be the small number of patients and lower mean age of the study population. Increased TyG indices and their association with plasma ADMA levels imply that this index may be used as a simple and practical measure to predict endothelial dysfunction in patient with KS.

hsCRP is a low-grade inflammation marker widely used as an indicator of increased cardiovascular risk (27, 28). In our study, no difference was found between the hsCRP levels of patients and the control group. This result is inconsistent with the reports of elevated hsCRP in patients with KS, published by Bojesen and cols. in the literature (4). We think that the reason for this discrepancy may be that our study population consists of individuals who were younger and did not have any chronic metabolic diseases.

There may be several limitations and strengths of the present study. First of all, owing the cross- sectional design of the study, it is not possible to make a mechanistic comment regarding the relationship between increased cardiometabolic risk and TyG index of patients with KS. In addition, the specific population of treatment-naïve, young patients with KS included in this study may not represent the general population with KS. Finally, low number of patients may be considered as another limitation. However, the homogeneous study population of similar age and sex and the lack of confounding factors, such as chronic metabolic diseases or medications, can be counted among the strengths of this study.

In conclusion, the present study revealed that TyG indices are higher in patients with KS than in healthy controls and are significantly correlated with endothelial dysfunction and insulin resistance markers. Furthermore, the TyG index is an independent predictor of endothelial dysfunction in patients with KS. These results imply that TyG index may be a useful and practical measure to detect endothelial dysfunction in patients with KS. However, prospective long-term studies with larger numbers are needed to evaluate the capability of the TyG index in predicting cardiometabolic risk in patients with KS.
